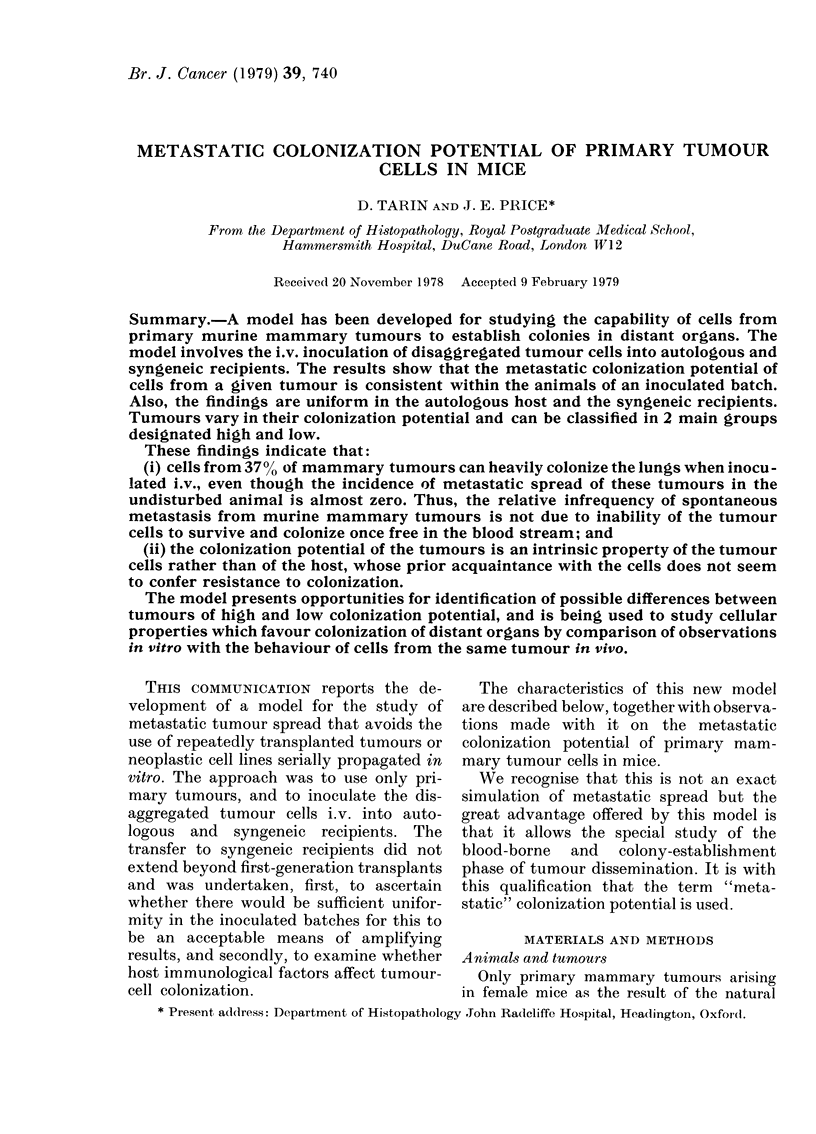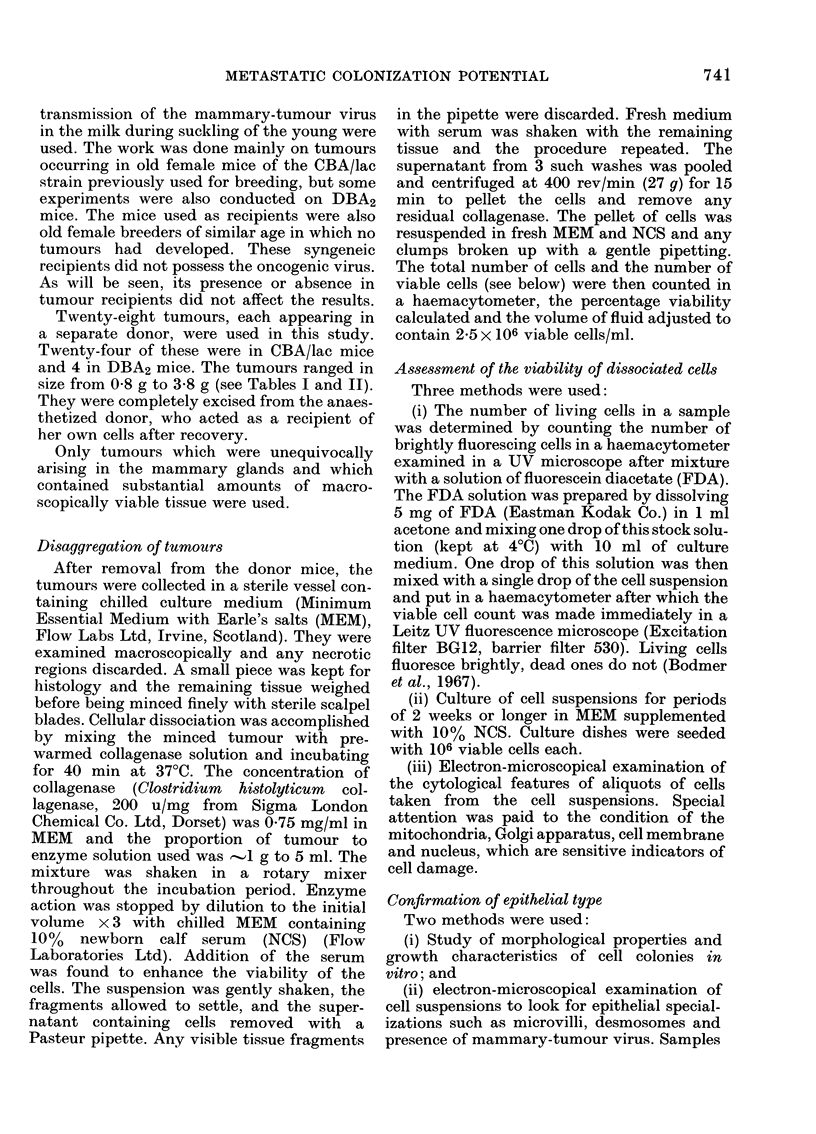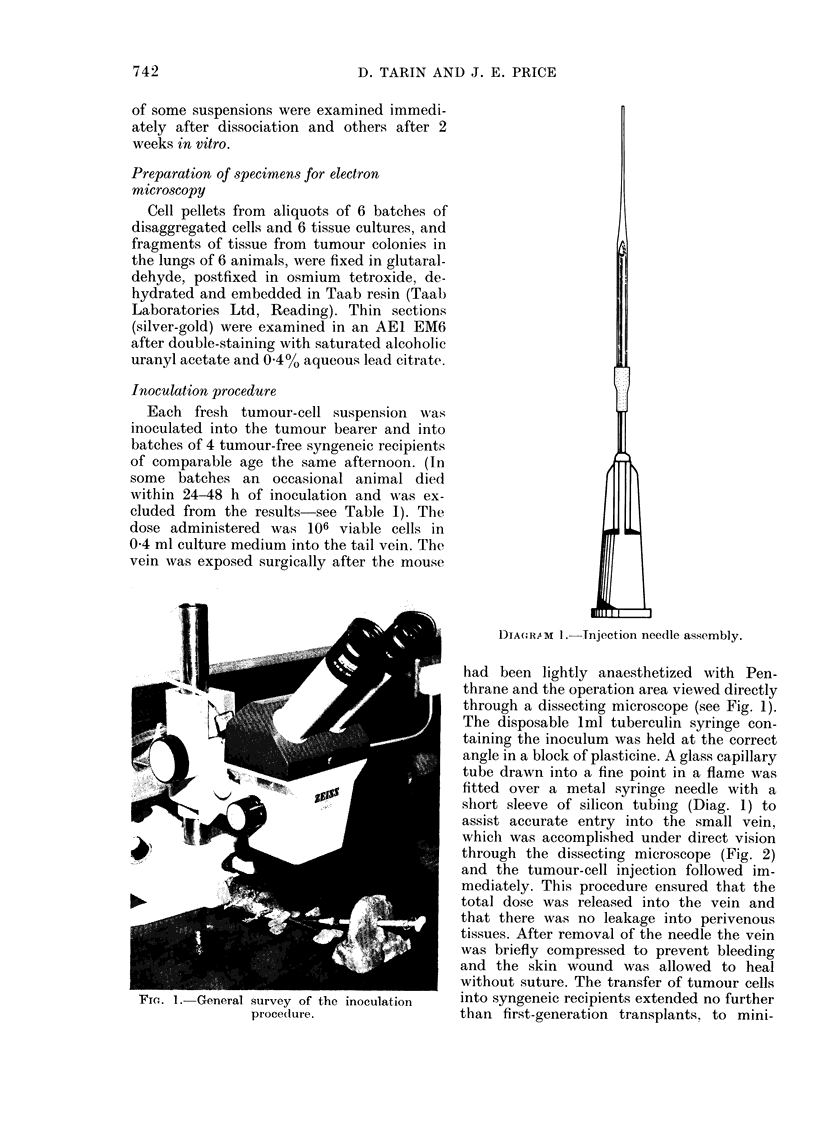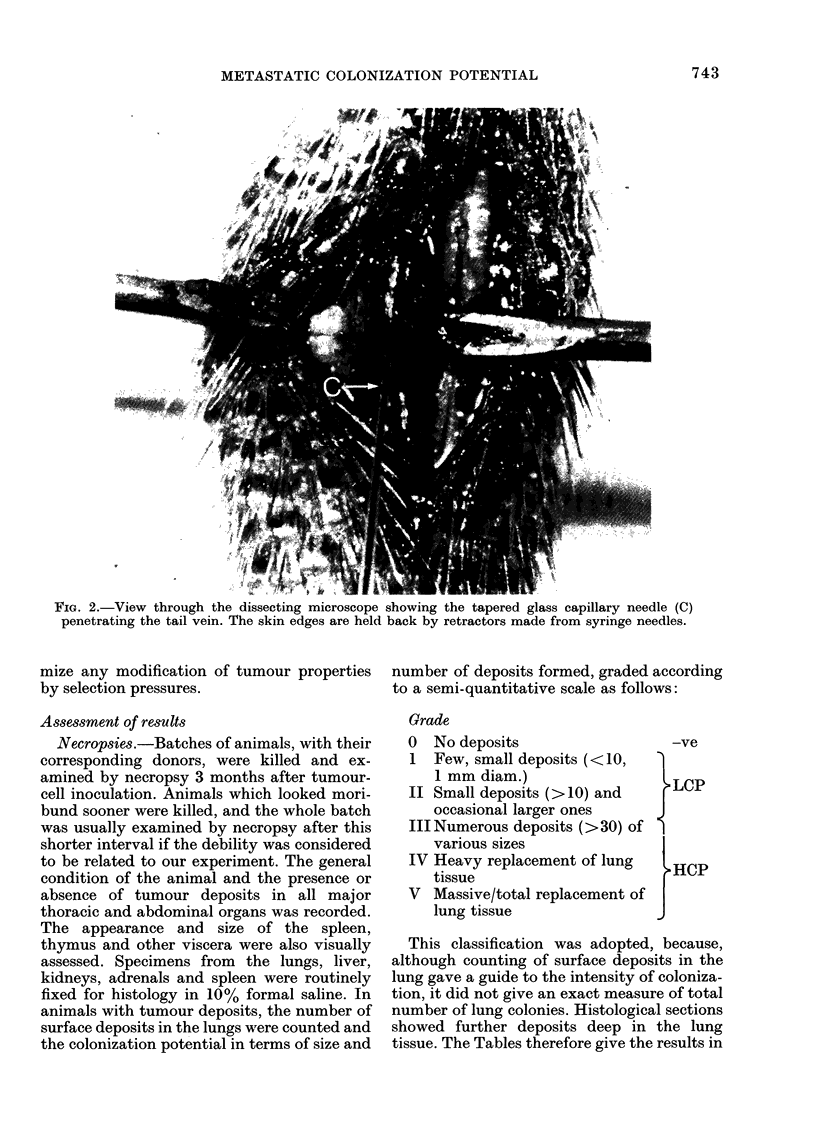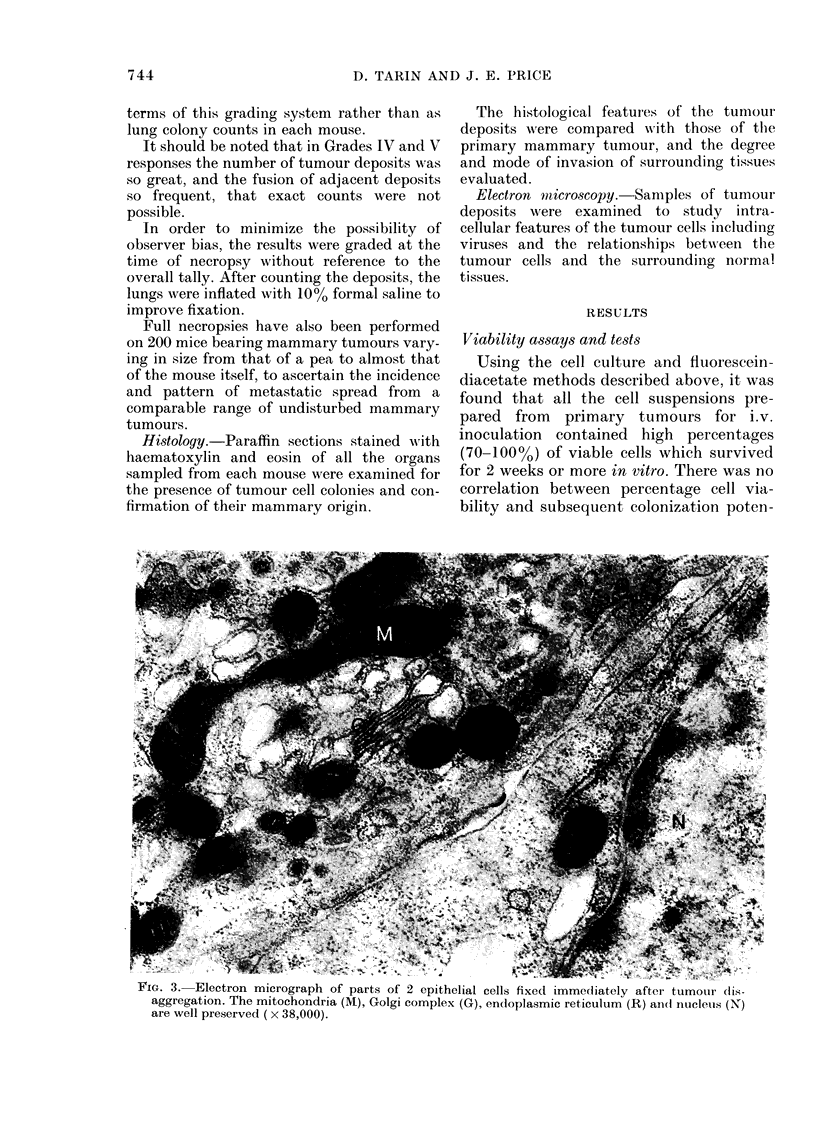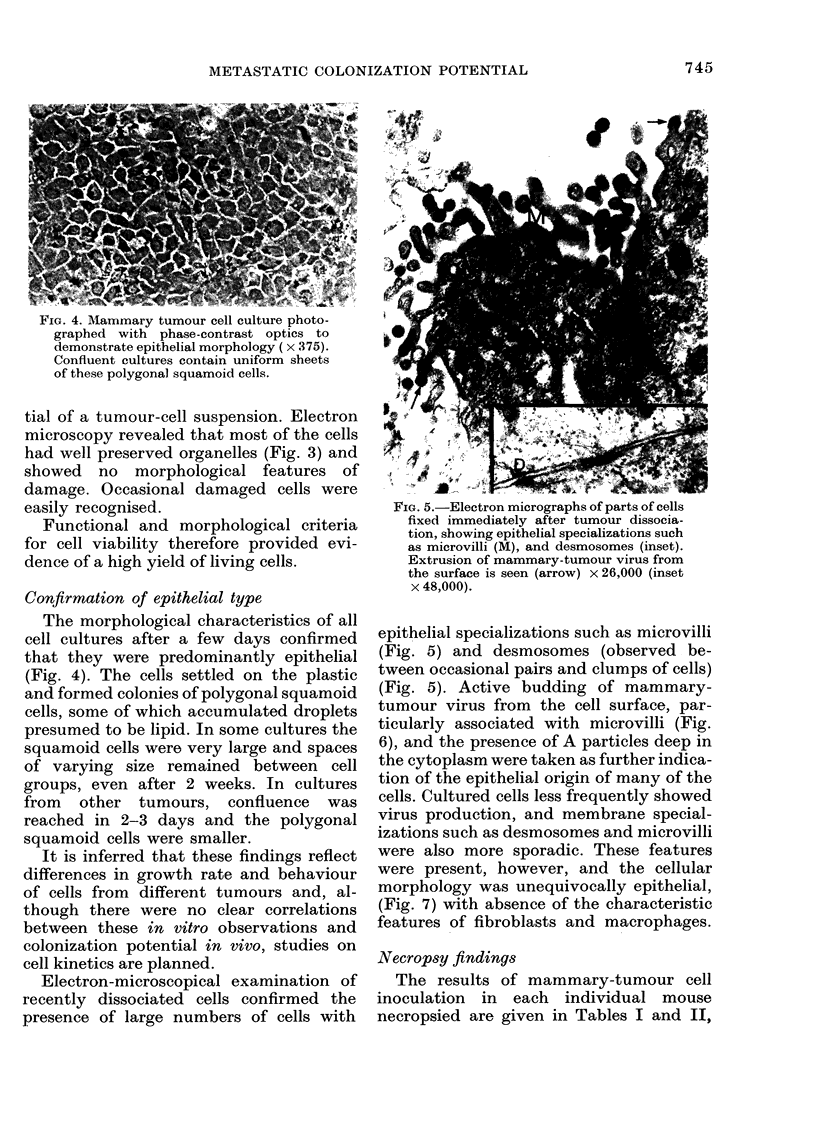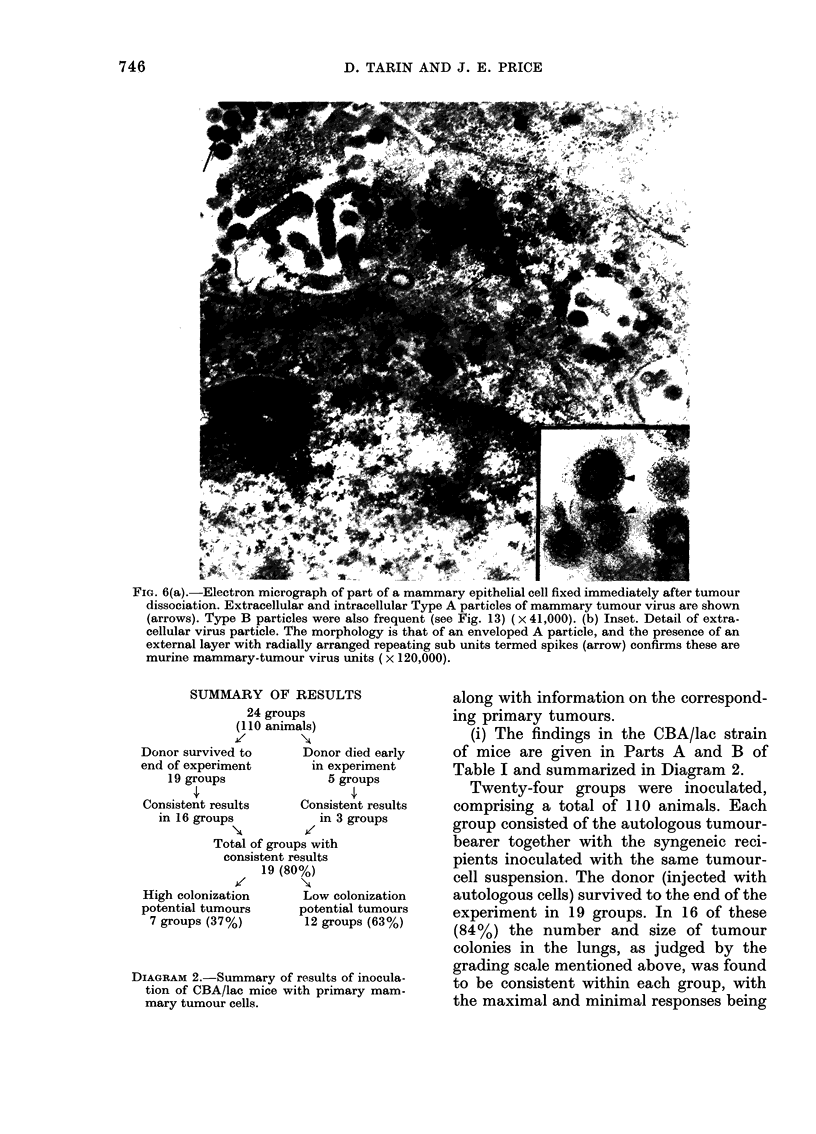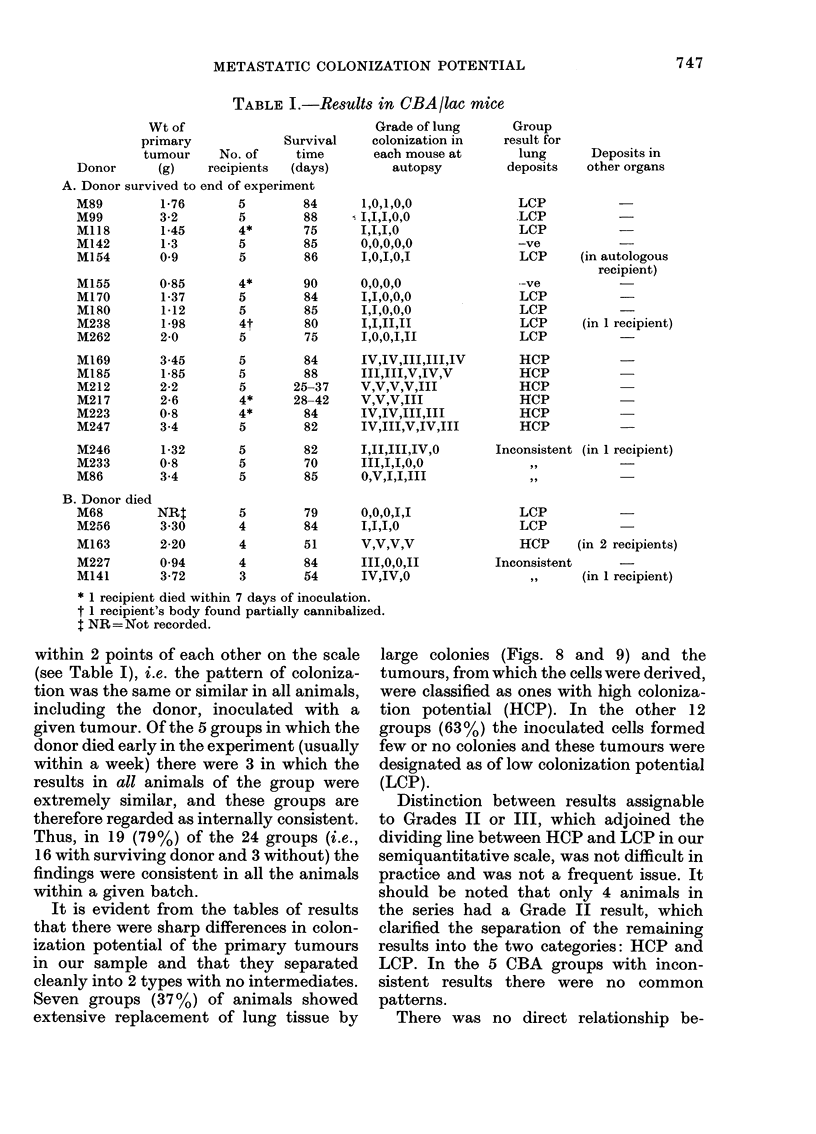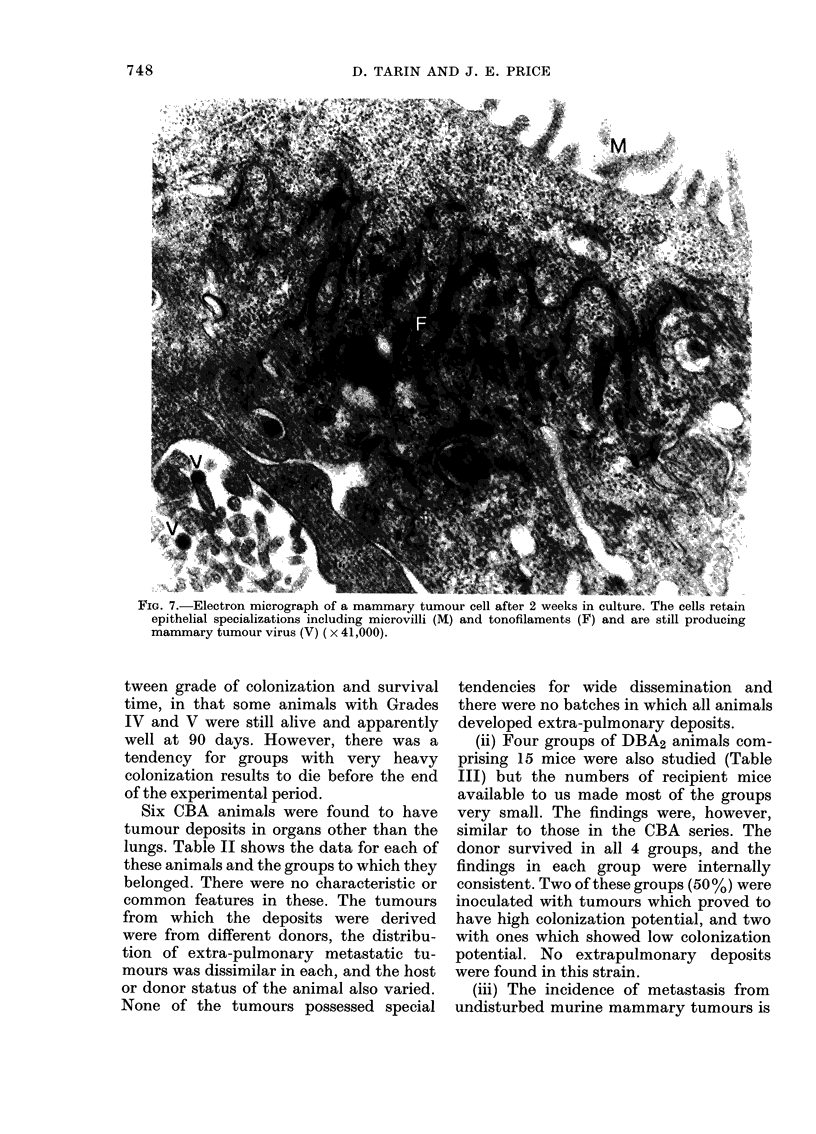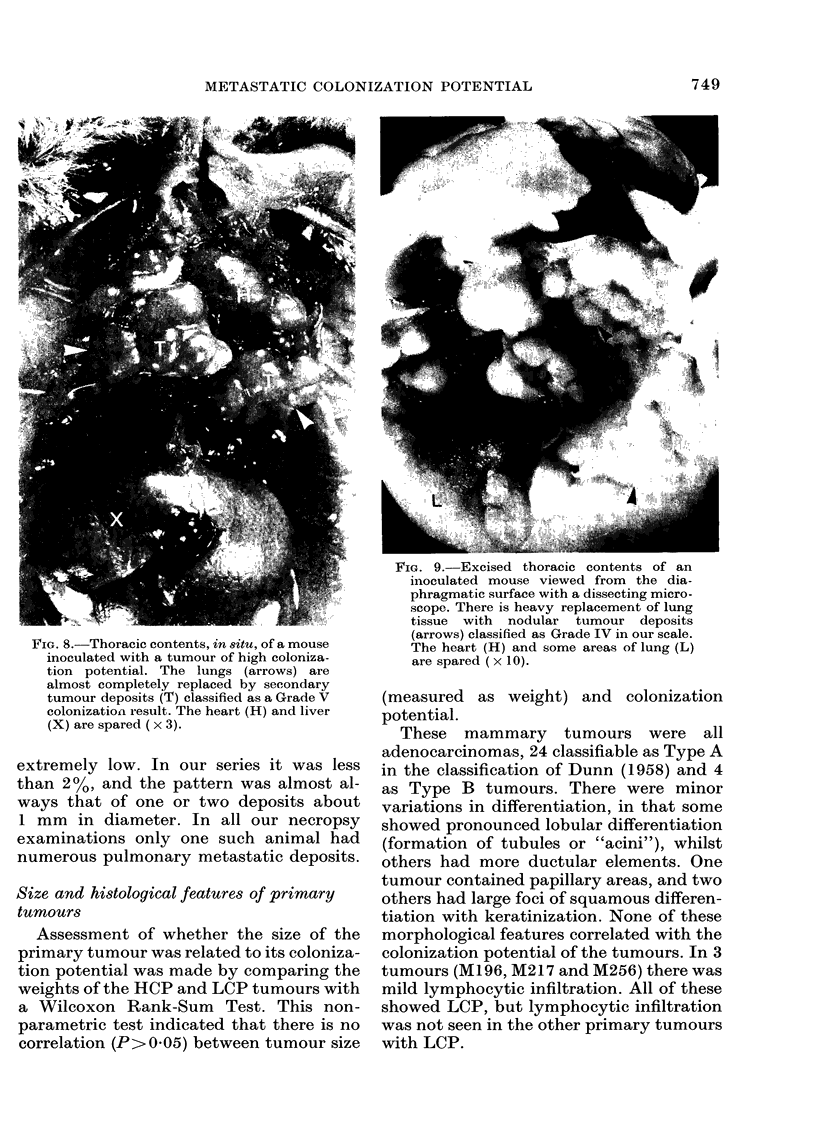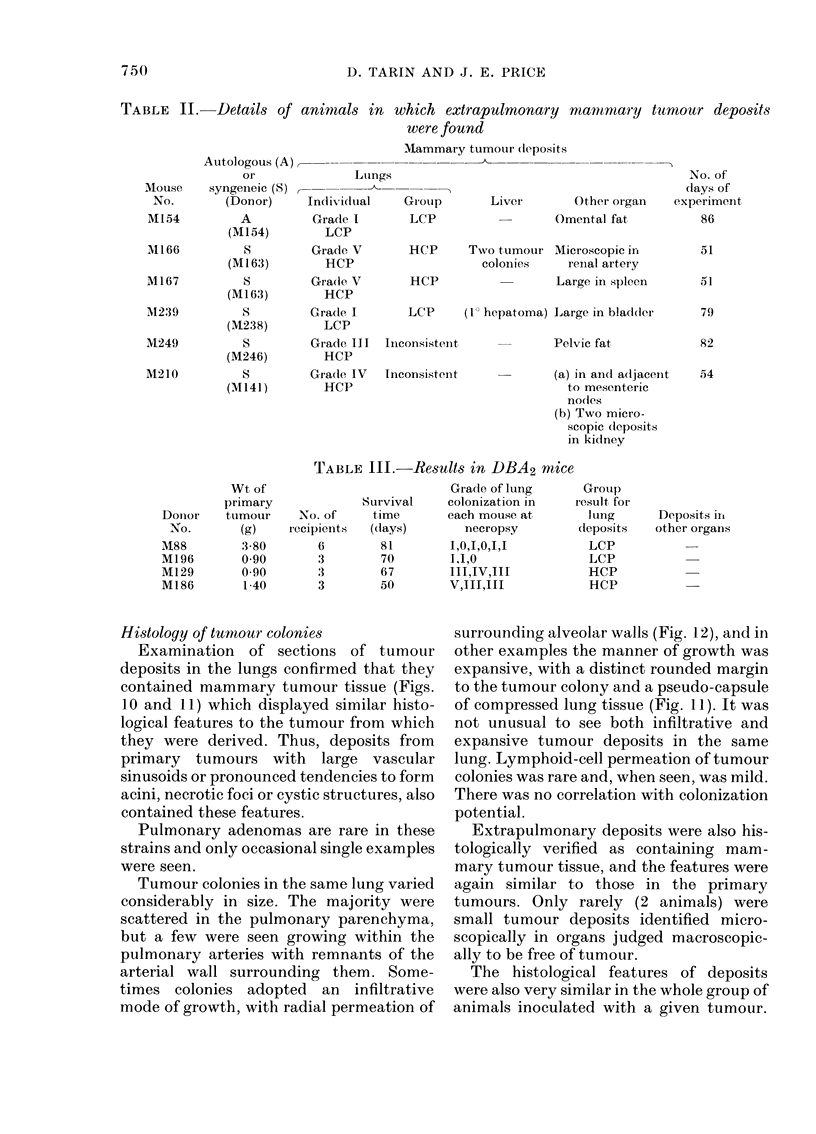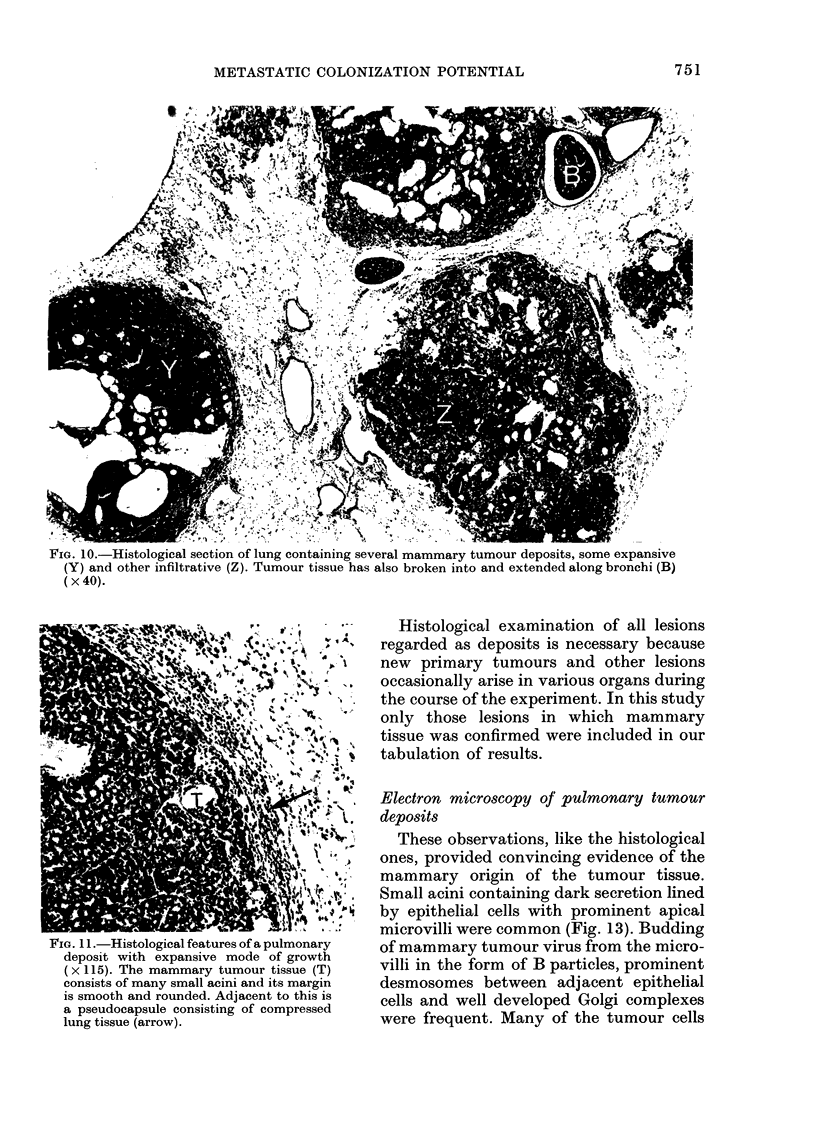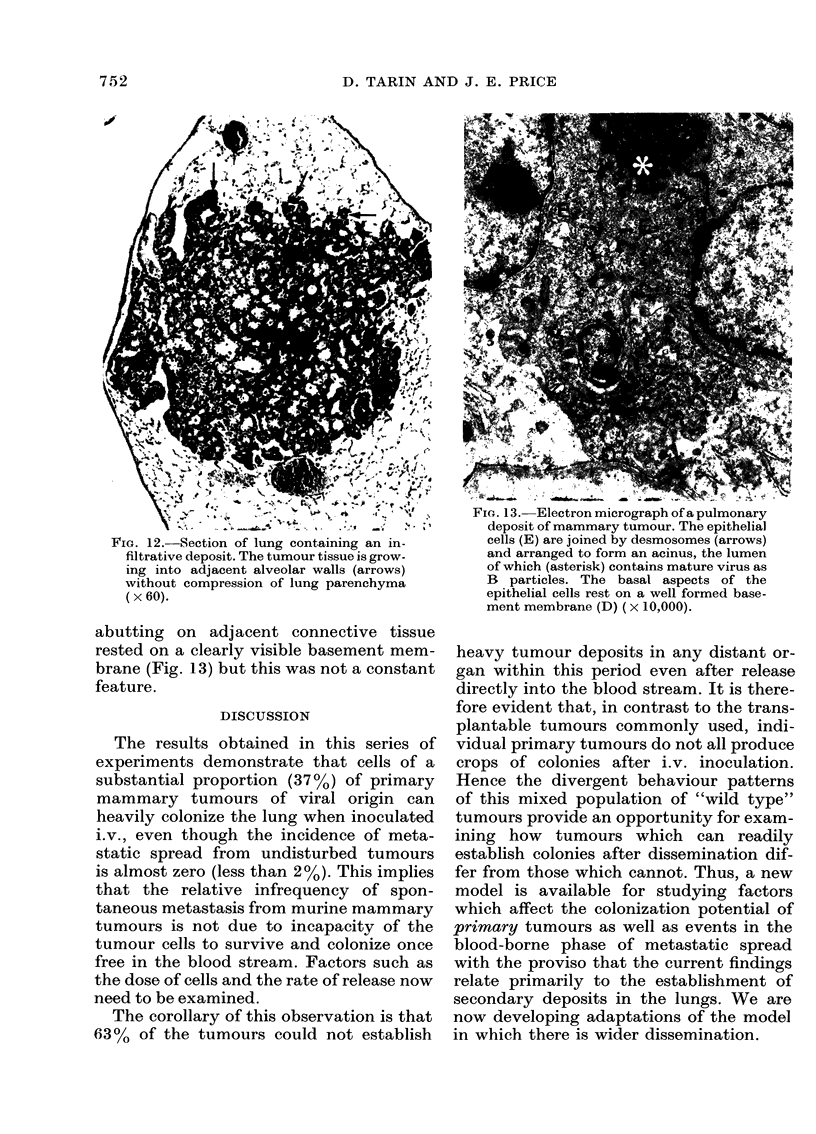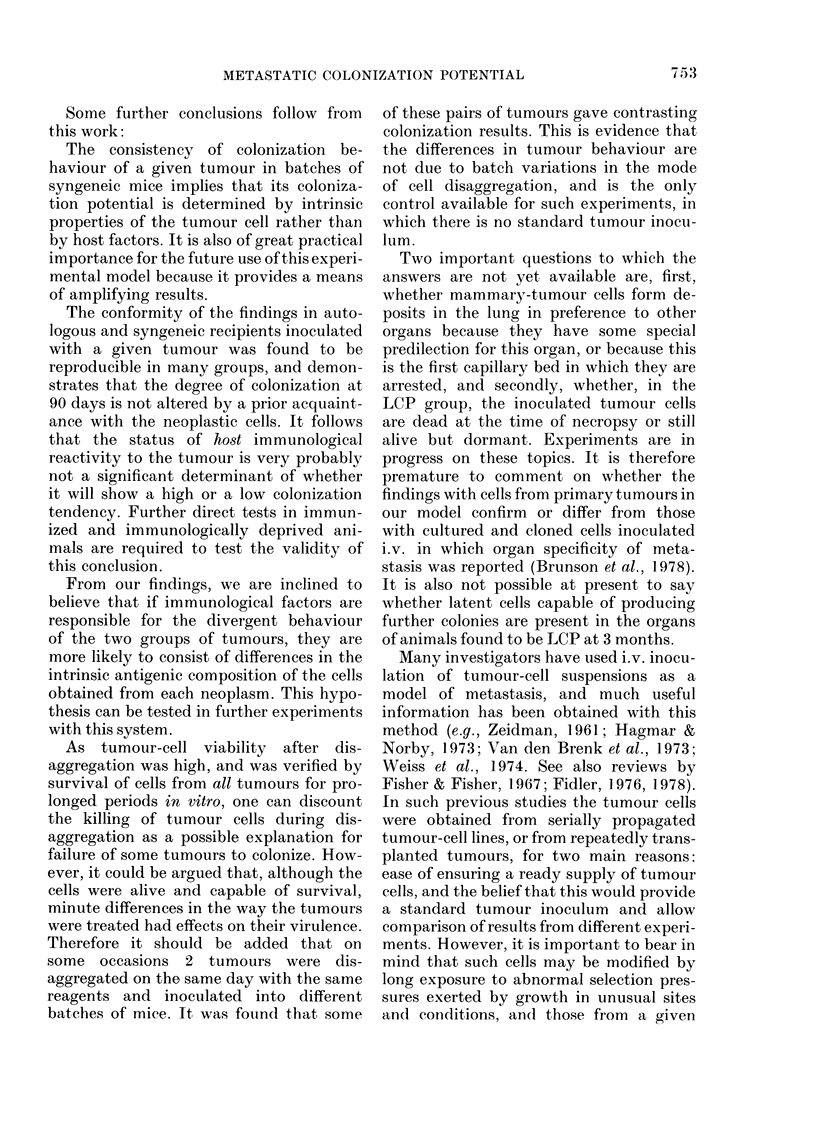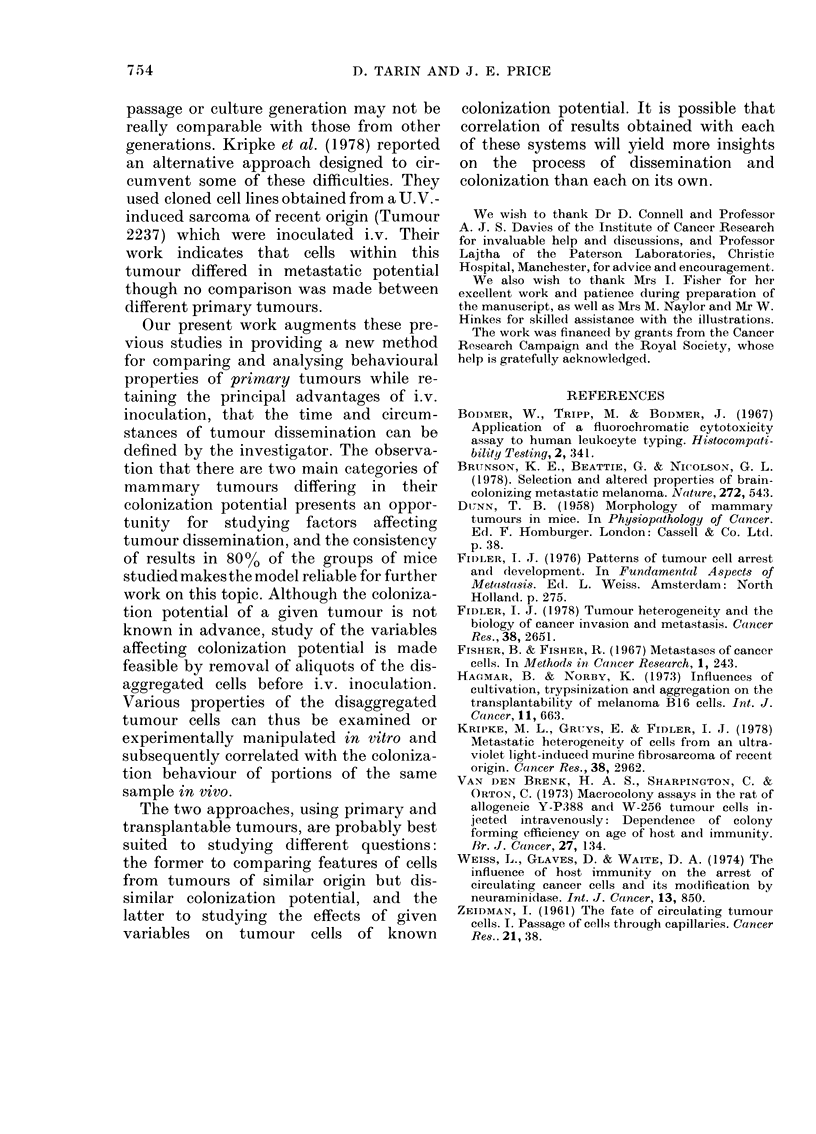# Metastatic colonization potential of primary tumour cells in mice.

**DOI:** 10.1038/bjc.1979.128

**Published:** 1979-06

**Authors:** D. Tarin, J. E. Price

## Abstract

**Images:**


*Br. J. Cancer* (1979) **39**, 740

### METASTATIC COLONIZATION POTENTIAL OF PRIMARY TUMOUR CELLS IN MICE

D. TARIN AND J. E. PRICE*

*From the Department of Histopathology, Royal Postgraduate Medical School, Hammersmith Hospital, DuCane Road, London W12*

Received 20 November 1978 Accepted 9 February 1979

**Summary.—A model has been developed for studying the capability of cells from primary murine mammary tumours to establish colonies in distant organs. The model involves the i.v. inoculation of disaggregated tumour cells into autologous and syngeneic recipients. The results show that the metastatic colonization potential of cells from a given tumour is consistent within the animals of an inoculated batch. Also, the findings are uniform in the autologous host and the syngeneic recipients. Tumours vary in their colonization potential and can be classified in 2 main groups designated high and low.**

**These findings indicate that:**

**(i) cells from 37% of mammary tumours can heavily colonize the lungs when inoculated i.v., even though the incidence of metastatic spread of these tumours in the undisturbed animal is almost zero. Thus, the relative infrequency of spontaneous metastasis from murine mammary tumours is not due to inability of the tumour cells to survive and colonize once free in the blood stream; and**

**(ii) the colonization potential of the tumours is an intrinsic property of the tumour cells rather than of the host, whose prior acquaintance with the cells does not seem to confer resistance to colonization.**

**The model presents opportunities for identification of possible differences between tumours of high and low colonization potential, and is being used to study cellular properties which favour colonization of distant organs by comparison of observations *in vitro* with the behaviour of cells from the same tumour *in vivo*.**

THIS COMMUNICATION reports the development of a model for the study of metastatic tumour spread that avoids the use of repeatedly transplanted tumours or neoplastic cell lines serially propagated *in vitro*. The approach was to use only primary tumours, and to inoculate the disaggregated tumour cells i.v. into autologous and syngeneic recipients. The transfer to syngeneic recipients did not extend beyond first-generation transplants and was undertaken, first, to ascertain whether there would be sufficient uniformity in the inoculated batches for this to be an acceptable means of amplifying results, and secondly, to examine whether host immunological factors affect tumour-cell colonization.

The characteristics of this new model are described below, together with observations made with it on the metastatic colonization potential of primary mammary tumour cells in mice.

We recognise that this is not an exact simulation of metastatic spread but the great advantage offered by this model is that it allows the special study of the blood-borne and colony-establishment phase of tumour dissemination. It is with this qualification that the term "metastatic" colonization potential is used.

### MATERIALS AND METHODS

### *Animals and tumours*

Only primary mammary tumours arising in female mice as the result of the natural

* Present address: Department of Histopathology John Radcliffe Hospital, Headington, Oxford.

transmission of the mammary-tumour virus in the milk during suckling of the young were used. The work was done mainly on tumours occurring in old female mice of the CBA/lac strain previously used for breeding, but some experiments were also conducted on $DBA_2$ mice. The mice used as recipients were also old female breeders of similar age in which no tumours had developed. These syngeneic recipients did not possess the oncogenic virus. As will be seen, its presence or absence in tumour recipients did not affect the results.

Twenty-eight tumours, each appearing in a separate donor, were used in this study. Twenty-four of these were in CBA/lac mice and 4 in $DBA_2$ mice. The tumours ranged in size from 0·8 g to 3·8 g (see Tables I and II). They were completely excised from the anaesthetized donor, who acted as a recipient of her own cells after recovery.

Only tumours which were unequivocally arising in the mammary glands and which contained substantial amounts of macroscopically viable tissue were used.

*Disaggregation of tumours*

After removal from the donor mice, the tumours were collected in a sterile vessel containing chilled culture medium (Minimum Essential Medium with Earle's salts (MEM), Flow Labs Ltd, Irvine, Scotland). They were examined macroscopically and any necrotic regions discarded. A small piece was kept for histology and the remaining tissue weighed before being minced finely with sterile scalpel blades. Cellular dissociation was accomplished by mixing the minced tumour with prewarmed collagenase solution and incubating for 40 min at 37°C. The concentration of collagenase (*Clostridium histolyticum* collagenase, 200 u/mg from Sigma London Chemical Co. Ltd, Dorset) was 0·75 mg/ml in MEM and the proportion of tumour to enzyme solution used was $\sim$1 g to 5 ml. The mixture was shaken in a rotary mixer throughout the incubation period. Enzyme action was stopped by dilution to the initial volume $\times 3$ with chilled MEM containing 10% newborn calf serum (NCS) (Flow Laboratories Ltd). Addition of the serum was found to enhance the viability of the cells. The suspension was gently shaken, the fragments allowed to settle, and the supernatant containing cells removed with a Pasteur pipette. Any visible tissue fragments in the pipette were discarded. Fresh medium with serum was shaken with the remaining tissue and the procedure repeated. The supernatant from 3 such washes was pooled and centrifuged at 400 rev/min (27 *g*) for 15 min to pellet the cells and remove any residual collagenase. The pellet of cells was resuspended in fresh MEM and NCS and any clumps broken up with a gentle pipetting. The total number of cells and the number of viable cells (see below) were then counted in a haemacytometer, the percentage viability calculated and the volume of fluid adjusted to contain $2{\cdot}5 \times 10^6$ viable cells/ml.

*Assessment of the viability of dissociated cells*

Three methods were used:

(i) The number of living cells in a sample was determined by counting the number of brightly fluorescing cells in a haemacytometer examined in a UV microscope after mixture with a solution of fluorescein diacetate (FDA). The FDA solution was prepared by dissolving 5 mg of FDA (Eastman Kodak Co.) in 1 ml acetone and mixing one drop of this stock solution (kept at 4°C) with 10 ml of culture medium. One drop of this solution was then mixed with a single drop of the cell suspension and put in a haemacytometer after which the viable cell count was made immediately in a Leitz UV fluorescence microscope (Excitation filter BG12, barrier filter 530). Living cells fluoresce brightly, dead ones do not (Bodmer *et al.*, 1967).

(ii) Culture of cell suspensions for periods of 2 weeks or longer in MEM supplemented with 10% NCS. Culture dishes were seeded with $10^6$ viable cells each.

(iii) Electron-microscopical examination of the cytological features of aliquots of cells taken from the cell suspensions. Special attention was paid to the condition of the mitochondria, Golgi apparatus, cell membrane and nucleus, which are sensitive indicators of cell damage.

*Confirmation of epithelial type*

Two methods were used:

(i) Study of morphological properties and growth characteristics of cell colonies *in vitro*; and

(ii) electron-microscopical examination of cell suspensions to look for epithelial specializations such as microvilli, desmosomes and presence of mammary-tumour virus. Samples of some suspensions were examined immediately after dissociation and others after 2 weeks *in vitro*.

*Preparation of specimens for electron microscopy*

Cell pellets from aliquots of 6 batches of disaggregated cells and 6 tissue cultures, and fragments of tissue from tumour colonies in the lungs of 6 animals, were fixed in glutaraldehyde, postfixed in osmium tetroxide, dehydrated and embedded in Taab resin (Taab Laboratories Ltd, Reading). Thin sections (silver-gold) were examined in an AE1 EM6 after double-staining with saturated alcoholic uranyl acetate and 0·4% aqueous lead citrate.

*Inoculation procedure*

Each fresh tumour-cell suspension was inoculated into the tumour bearer and into batches of 4 tumour-free syngeneic recipients of comparable age the same afternoon. (In some batches an occasional animal died within 24–48 h of inoculation and was excluded from the results—see Table I). The dose administered was $10^6$ viable cells in 0·4 ml culture medium into the tail vein. The vein was exposed surgically after the mouse had been lightly anaesthetized with Penthrane and the operation area viewed directly through a dissecting microscope (see Fig. 1). The disposable 1ml tuberculin syringe containing the inoculum was held at the correct angle in a block of plasticine. A glass capillary tube drawn into a fine point in a flame was fitted over a metal syringe needle with a short sleeve of silicon tubing (Diag. 1) to assist accurate entry into the small vein, which was accomplished under direct vision through the dissecting microscope (Fig. 2) and the tumour-cell injection followed immediately. This procedure ensured that the total dose was released into the vein and that there was no leakage into perivenous tissues. After removal of the needle the vein was briefly compressed to prevent bleeding and the skin wound was allowed to heal without suture. The transfer of tumour cells into syngeneic recipients extended no further than first-generation transplants, to mini-

DIAGRAM 1.—Injection needle assembly.

FIG. 1.—General survey of the inoculation procedure.

FIG. 2.—View through the dissecting microscope showing the tapered glass capillary needle (C) penetrating the tail vein. The skin edges are held back by retractors made from syringe needles.

mize any modification of tumour properties by selection pressures.

*Assessment of results*

*Necropsies.*—Batches of animals, with their corresponding donors, were killed and examined by necropsy 3 months after tumour-cell inoculation. Animals which looked moribund sooner were killed, and the whole batch was usually examined by necropsy after this shorter interval if the debility was considered to be related to our experiment. The general condition of the animal and the presence or absence of tumour deposits in all major thoracic and abdominal organs was recorded. The appearance and size of the spleen, thymus and other viscera were also visually assessed. Specimens from the lungs, liver, kidneys, adrenals and spleen were routinely fixed for histology in 10% formal saline. In animals with tumour deposits, the number of surface deposits in the lungs were counted and the colonization potential in terms of size and number of deposits formed, graded according to a semi-quantitative scale as follows:

| *Grade* | | |
|---|---|---|
| 0 | No deposits | –ve |
| 1 | Few, small deposits (<10, 1 mm diam.) | LCP |
| II | Small deposits (>10) and occasional larger ones | LCP |
| III | Numerous deposits (>30) of various sizes | HCP |
| IV | Heavy replacement of lung tissue | HCP |
| V | Massive/total replacement of lung tissue | HCP |

This classification was adopted, because, although counting of surface deposits in the lung gave a guide to the intensity of colonization, it did not give an exact measure of total number of lung colonies. Histological sections showed further deposits deep in the lung tissue. The Tables therefore give the results in terms of this grading system rather than as lung colony counts in each mouse.

It should be noted that in Grades IV and V responses the number of tumour deposits was so great, and the fusion of adjacent deposits so frequent, that exact counts were not possible.

In order to minimize the possibility of observer bias, the results were graded at the time of necropsy without reference to the overall tally. After counting the deposits, the lungs were inflated with 10% formal saline to improve fixation.

Full necropsies have also been performed on 200 mice bearing mammary tumours varying in size from that of a pea to almost that of the mouse itself, to ascertain the incidence and pattern of metastatic spread from a comparable range of undisturbed mammary tumours.

*Histology*.—Paraffin sections stained with haematoxylin and eosin of all the organs sampled from each mouse were examined for the presence of tumour cell colonies and confirmation of their mammary origin.

The histological features of the tumour deposits were compared with those of the primary mammary tumour, and the degree and mode of invasion of surrounding tissues evaluated.

*Electron microscopy*.—Samples of tumour deposits were examined to study intracellular features of the tumour cells including viruses and the relationships between the tumour cells and the surrounding normal tissues.

### RESULTS

### *Viability assays and tests*

Using the cell culture and fluorescein-diacetate methods described above, it was found that all the cell suspensions prepared from primary tumours for i.v. inoculation contained high percentages (70–100%) of viable cells which survived for 2 weeks or more *in vitro*. There was no correlation between percentage cell viability and subsequent colonization poten-

FIG. 3.—Electron micrograph of parts of 2 epithelial cells fixed immediately after tumour disaggregation. The mitochondria (M), Golgi complex (G), endoplasmic reticulum (R) and nucleus (N) are well preserved (× 38,000).

FIG. 4. Mammary tumour cell culture photographed with phase-contrast optics to demonstrate epithelial morphology (×375). Confluent cultures contain uniform sheets of these polygonal squamoid cells.

tial of a tumour-cell suspension. Electron microscopy revealed that most of the cells had well preserved organelles (Fig. 3) and showed no morphological features of damage. Occasional damaged cells were easily recognised.

Functional and morphological criteria for cell viability therefore provided evidence of a high yield of living cells.

*Confirmation of epithelial type*

The morphological characteristics of all cell cultures after a few days confirmed that they were predominantly epithelial (Fig. 4). The cells settled on the plastic and formed colonies of polygonal squamoid cells, some of which accumulated droplets presumed to be lipid. In some cultures the squamoid cells were very large and spaces of varying size remained between cell groups, even after 2 weeks. In cultures from other tumours, confluence was reached in 2–3 days and the polygonal squamoid cells were smaller.

It is inferred that these findings reflect differences in growth rate and behaviour of cells from different tumours and, although there were no clear correlations between these *in vitro* observations and colonization potential *in vivo*, studies on cell kinetics are planned.

Electron-microscopical examination of recently dissociated cells confirmed the presence of large numbers of cells with

FIG. 5.—Electron micrographs of parts of cells fixed immediately after tumour dissociation, showing epithelial specializations such as microvilli (M), and desmosomes (inset). Extrusion of mammary-tumour virus from the surface is seen (arrow) ×26,000 (inset ×48,000).

epithelial specializations such as microvilli (Fig. 5) and desmosomes (observed between occasional pairs and clumps of cells) (Fig. 5). Active budding of mammary-tumour virus from the cell surface, particularly associated with microvilli (Fig. 6), and the presence of A particles deep in the cytoplasm were taken as further indication of the epithelial origin of many of the cells. Cultured cells less frequently showed virus production, and membrane specializations such as desmosomes and microvilli were also more sporadic. These features were present, however, and the cellular morphology was unequivocally epithelial, (Fig. 7) with absence of the characteristic features of fibroblasts and macrophages.

*Necropsy findings*

The results of mammary-tumour cell inoculation in each individual mouse necropsied are given in Tables I and II,

FIG. 6(a).—Electron micrograph of part of a mammary epithelial cell fixed immediately after tumour dissociation. Extracellular and intracellular Type A particles of mammary tumour virus are shown (arrows). Type B particles were also frequent (see Fig. 13) (×41,000). (b) Inset. Detail of extracellular virus particle. The morphology is that of an enveloped A particle, and the presence of an external layer with radially arranged repeating sub units termed spikes (arrow) confirms these are murine mammary-tumour virus units (×120,000).

SUMMARY OF RESULTS

24 groups
(110 animals)
↙ ↘

| Donor survived to end of experiment 19 groups | Donor died early in experiment 5 groups |
|---|---|
| ↓ | ↓ |
| Consistent results in 16 groups ↘ | ↙ Consistent results in 3 groups |

Total of groups with consistent results
19 (80%)
↙ ↘

| High colonization potential tumours 7 groups (37%) | Low colonization potential tumours 12 groups (63%) |
|---|---|

DIAGRAM 2.—Summary of results of inoculation of CBA/lac mice with primary mammary tumour cells.

along with information on the corresponding primary tumours.

(i) The findings in the CBA/lac strain of mice are given in Parts A and B of Table I and summarized in Diagram 2.

Twenty-four groups were inoculated, comprising a total of 110 animals. Each group consisted of the autologous tumour-bearer together with the syngeneic recipients inoculated with the same tumour-cell suspension. The donor (injected with autologous cells) survived to the end of the experiment in 19 groups. In 16 of these (84%) the number and size of tumour colonies in the lungs, as judged by the grading scale mentioned above, was found to be consistent within each group, with the maximal and minimal responses being

TABLE I.—*Results in CBA/lac mice*

| Donor | Wt of primary tumour (g) | No. of recipients | Survival time (days) | Grade of lung colonization in each mouse at autopsy | Group result for lung deposits | Deposits in other organs |
|---|---|---|---|---|---|---|
| A. Donor survived to end of experiment | | | | | | |
| M89 | 1·76 | 5 | 84 | 1,0,1,0,0 | LCP | — |
| M99 | 3·2 | 5 | 88 | I,I,I,0,0 | LCP | — |
| M118 | 1·45 | 4* | 75 | I,I,I,0 | LCP | — |
| M142 | 1·3 | 5 | 85 | 0,0,0,0,0 | –ve | — |
| M154 | 0·9 | 5 | 86 | I,0,I,0,I | LCP | (in autologous recipient) |
| M155 | 0·85 | 4* | 90 | 0,0,0,0 | –ve | — |
| M170 | 1·37 | 5 | 84 | I,I,0,0,0 | LCP | — |
| M180 | 1·12 | 5 | 85 | I,I,0,0,0 | LCP | — |
| M238 | 1·98 | 4† | 80 | I,I,II,II | LCP | (in 1 recipient) |
| M262 | 2·0 | 5 | 75 | I,0,0,I,II | LCP | — |
| M169 | 3·45 | 5 | 84 | IV,IV,III,III,IV | HCP | — |
| M185 | 1·85 | 5 | 88 | III,III,V,IV,V | HCP | — |
| M212 | 2·2 | 5 | 25–37 | V,V,V,V,III | HCP | — |
| M217 | 2·6 | 4* | 28–42 | V,V,V,III | HCP | — |
| M223 | 0·8 | 4* | 84 | IV,IV,III,III | HCP | — |
| M247 | 3·4 | 5 | 82 | IV,III,V,IV,III | HCP | — |
| M246 | 1·32 | 5 | 82 | I,II,III,IV,0 | Inconsistent | (in 1 recipient) |
| M233 | 0·8 | 5 | 70 | III,I,I,0,0 | ,, | — |
| M86 | 3·4 | 5 | 85 | 0,V,I,I,III | ,, | — |
| B. Donor died | | | | | | |
| M68 | NR‡ | 5 | 79 | 0,0,0,I,I | LCP | — |
| M256 | 3·30 | 4 | 84 | I,I,I,0 | LCP | — |
| M163 | 2·20 | 4 | 51 | V,V,V,V | HCP | (in 2 recipients) |
| M227 | 0·94 | 4 | 84 | III,0,0,II | Inconsistent | — |
| M141 | 3·72 | 3 | 54 | IV,IV,0 | ,, | (in 1 recipient) |

* 1 recipient died within 7 days of inoculation.
† 1 recipient's body found partially cannibalized.
‡ NR = Not recorded.

within 2 points of each other on the scale (see Table I), *i.e.* the pattern of colonization was the same or similar in all animals, including the donor, inoculated with a given tumour. Of the 5 groups in which the donor died early in the experiment (usually within a week) there were 3 in which the results in *all* animals of the group were extremely similar, and these groups are therefore regarded as internally consistent. Thus, in 19 (79%) of the 24 groups (*i.e.*, 16 with surviving donor and 3 without) the findings were consistent in all the animals within a given batch.

It is evident from the tables of results that there were sharp differences in colonization potential of the primary tumours in our sample and that they separated cleanly into 2 types with no intermediates. Seven groups (37%) of animals showed extensive replacement of lung tissue by large colonies (Figs. 8 and 9) and the tumours, from which the cells were derived, were classified as ones with high colonization potential (HCP). In the other 12 groups (63%) the inoculated cells formed few or no colonies and these tumours were designated as of low colonization potential (LCP).

Distinction between results assignable to Grades II or III, which adjoined the dividing line between HCP and LCP in our semiquantitative scale, was not difficult in practice and was not a frequent issue. It should be noted that only 4 animals in the series had a Grade II result, which clarified the separation of the remaining results into the two categories: HCP and LCP. In the 5 CBA groups with inconsistent results there were no common patterns.

There was no direct relationship be-

FIG. 7.—Electron micrograph of a mammary tumour cell after 2 weeks in culture. The cells retain epithelial specializations including microvilli (M) and tonofilaments (F) and are still producing mammary tumour virus (V) ( × 41,000).

tween grade of colonization and survival time, in that some animals with Grades IV and V were still alive and apparently well at 90 days. However, there was a tendency for groups with very heavy colonization results to die before the end of the experimental period.

Six CBA animals were found to have tumour deposits in organs other than the lungs. Table II shows the data for each of these animals and the groups to which they belonged. There were no characteristic or common features in these. The tumours from which the deposits were derived were from different donors, the distribution of extra-pulmonary metastatic tumours was dissimilar in each, and the host or donor status of the animal also varied. None of the tumours possessed special tendencies for wide dissemination and there were no batches in which all animals developed extra-pulmonary deposits.

(ii) Four groups of $DBA_2$ animals comprising 15 mice were also studied (Table III) but the numbers of recipient mice available to us made most of the groups very small. The findings were, however, similar to those in the CBA series. The donor survived in all 4 groups, and the findings in each group were internally consistent. Two of these groups (50%) were inoculated with tumours which proved to have high colonization potential, and two with ones which showed low colonization potential. No extrapulmonary deposits were found in this strain.

(iii) The incidence of metastasis from undisturbed murine mammary tumours is

FIG. 8.—Thoracic contents, *in situ*, of a mouse inoculated with a tumour of high colonization potential. The lungs (arrows) are almost completely replaced by secondary tumour deposits (T) classified as a Grade V colonization result. The heart (H) and liver (X) are spared (× 3).

FIG. 9.—Excised thoracic contents of an inoculated mouse viewed from the diaphragmatic surface with a dissecting microscope. There is heavy replacement of lung tissue with nodular tumour deposits (arrows) classified as Grade IV in our scale. The heart (H) and some areas of lung (L) are spared (× 10).

extremely low. In our series it was less than 2%, and the pattern was almost always that of one or two deposits about 1 mm in diameter. In all our necropsy examinations only one such animal had numerous pulmonary metastatic deposits.

*Size and histological features of primary tumours*

Assessment of whether the size of the primary tumour was related to its colonization potential was made by comparing the weights of the HCP and LCP tumours with a Wilcoxon Rank-Sum Test. This non-parametric test indicated that there is no correlation ($P > 0{\cdot}05$) between tumour size (measured as weight) and colonization potential.

These mammary tumours were all adenocarcinomas, 24 classifiable as Type A in the classification of Dunn (1958) and 4 as Type B tumours. There were minor variations in differentiation, in that some showed pronounced lobular differentiation (formation of tubules or "acini"), whilst others had more ductular elements. One tumour contained papillary areas, and two others had large foci of squamous differentiation with keratinization. None of these morphological features correlated with the colonization potential of the tumours. In 3 tumours (M196, M217 and M256) there was mild lymphocytic infiltration. All of these showed LCP, but lymphocytic infiltration was not seen in the other primary tumours with LCP.

TABLE II.—*Details of animals in which extrapulmonary mammary tumour deposits were found*

| Mouse No. | Autologous (A) or syngeneic (S) (Donor) | Mammary tumour deposits: Lungs: Individual | Group | Liver | Other organ | No. of days of experiment |
|---|---|---|---|---|---|---|
| M154 | A (M154) | Grade I LCP | LCP | — | Omental fat | 86 |
| M166 | S (M163) | Grade V HCP | HCP | Two tumour colonies | Microscopic in renal artery | 51 |
| M167 | S (M163) | Grade V HCP | HCP | — | Large in spleen | 51 |
| M239 | S (M238) | Grade I LCP | LCP | (1° hepatoma) | Large in bladder | 79 |
| M249 | S (M246) | Grade III HCP | Inconsistent | — | Pelvic fat | 82 |
| M210 | S (M141) | Grade IV HCP | Inconsistent | — | (a) in and adjacent to mesenteric nodes (b) Two microscopic deposits in kidney | 54 |

TABLE III.—*Results in $DBA_2$ mice*

| Donor No. | Wt of primary tumour (g) | No. of recipients | Survival time (days) | Grade of lung colonization in each mouse at necropsy | Group result for lung deposits | Deposits in other organs |
|---|---|---|---|---|---|---|
| M88 | 3·80 | 6 | 81 | I,0,I,0,I,I | LCP | — |
| M196 | 0·90 | 3 | 70 | I,I,0 | LCP | — |
| M129 | 0·90 | 3 | 67 | III,IV,III | HCP | — |
| M186 | 1·40 | 3 | 50 | V,III,III | HCP | — |

*Histology of tumour colonies*

Examination of sections of tumour deposits in the lungs confirmed that they contained mammary tumour tissue (Figs. 10 and 11) which displayed similar histological features to the tumour from which they were derived. Thus, deposits from primary tumours with large vascular sinusoids or pronounced tendencies to form acini, necrotic foci or cystic structures, also contained these features.

Pulmonary adenomas are rare in these strains and only occasional single examples were seen.

Tumour colonies in the same lung varied considerably in size. The majority were scattered in the pulmonary parenchyma, but a few were seen growing within the pulmonary arteries with remnants of the arterial wall surrounding them. Sometimes colonies adopted an infiltrative mode of growth, with radial permeation of surrounding alveolar walls (Fig. 12), and in other examples the manner of growth was expansive, with a distinct rounded margin to the tumour colony and a pseudo-capsule of compressed lung tissue (Fig. 11). It was not unusual to see both infiltrative and expansive tumour deposits in the same lung. Lymphoid-cell permeation of tumour colonies was rare and, when seen, was mild. There was no correlation with colonization potential.

Extrapulmonary deposits were also histologically verified as containing mammary tumour tissue, and the features were again similar to those in the primary tumours. Only rarely (2 animals) were small tumour deposits identified microscopically in organs judged macroscopically to be free of tumour.

The histological features of deposits were also very similar in the whole group of animals inoculated with a given tumour.

FIG. 10.—Histological section of lung containing several mammary tumour deposits, some expansive (Y) and other infiltrative (Z). Tumour tissue has also broken into and extended along bronchi (B) (×40).

FIG. 11.—Histological features of a pulmonary deposit with expansive mode of growth (×115). The mammary tumour tissue (T) consists of many small acini and its margin is smooth and rounded. Adjacent to this is a pseudocapsule consisting of compressed lung tissue (arrow).

Histological examination of all lesions regarded as deposits is necessary because new primary tumours and other lesions occasionally arise in various organs during the course of the experiment. In this study only those lesions in which mammary tissue was confirmed were included in our tabulation of results.

*Electron microscopy of pulmonary tumour deposits*

These observations, like the histological ones, provided convincing evidence of the mammary origin of the tumour tissue. Small acini containing dark secretion lined by epithelial cells with prominent apical microvilli were common (Fig. 13). Budding of mammary tumour virus from the microvilli in the form of B particles, prominent desmosomes between adjacent epithelial cells and well developed Golgi complexes were frequent. Many of the tumour cells

FIG. 12.—Section of lung containing an infiltrative deposit. The tumour tissue is growing into adjacent alveolar walls (arrows) without compression of lung parenchyma ($\times$ 60).

FIG. 13.—Electron micrograph of a pulmonary deposit of mammary tumour. The epithelial cells (E) are joined by desmosomes (arrows) and arranged to form an acinus, the lumen of which (asterisk) contains mature virus as B particles. The basal aspects of the epithelial cells rest on a well formed basement membrane (D) ($\times$ 10,000).

abutting on adjacent connective tissue rested on a clearly visible basement membrane (Fig. 13) but this was not a constant feature.

DISCUSSION

The results obtained in this series of experiments demonstrate that cells of a substantial proportion (37%) of primary mammary tumours of viral origin can heavily colonize the lung when inoculated i.v., even though the incidence of metastatic spread from undisturbed tumours is almost zero (less than 2%). This implies that the relative infrequency of spontaneous metastasis from murine mammary tumours is not due to incapacity of the tumour cells to survive and colonize once free in the blood stream. Factors such as the dose of cells and the rate of release now need to be examined.

The corollary of this observation is that 63% of the tumours could not establish heavy tumour deposits in any distant organ within this period even after release directly into the blood stream. It is therefore evident that, in contrast to the transplantable tumours commonly used, individual primary tumours do not all produce crops of colonies after i.v. inoculation. Hence the divergent behaviour patterns of this mixed population of "wild type" tumours provide an opportunity for examining how tumours which can readily establish colonies after dissemination differ from those which cannot. Thus, a new model is available for studying factors which affect the colonization potential of *primary* tumours as well as events in the blood-borne phase of metastatic spread with the proviso that the current findings relate primarily to the establishment of secondary deposits in the lungs. We are now developing adaptations of the model in which there is wider dissemination.

Some further conclusions follow from this work:

The consistency of colonization behaviour of a given tumour in batches of syngeneic mice implies that its colonization potential is determined by intrinsic properties of the tumour cell rather than by host factors. It is also of great practical importance for the future use of this experimental model because it provides a means of amplifying results.

The conformity of the findings in autologous and syngeneic recipients inoculated with a given tumour was found to be reproducible in many groups, and demonstrates that the degree of colonization at 90 days is not altered by a prior acquaintance with the neoplastic cells. It follows that the status of *host* immunological reactivity to the tumour is very probably not a significant determinant of whether it will show a high or a low colonization tendency. Further direct tests in immunized and immunologically deprived animals are required to test the validity of this conclusion.

From our findings, we are inclined to believe that if immunological factors are responsible for the divergent behaviour of the two groups of tumours, they are more likely to consist of differences in the intrinsic antigenic composition of the cells obtained from each neoplasm. This hypothesis can be tested in further experiments with this system.

As tumour-cell viability after disaggregation was high, and was verified by survival of cells from *all* tumours for prolonged periods *in vitro*, one can discount the killing of tumour cells during disaggregation as a possible explanation for failure of some tumours to colonize. However, it could be argued that, although the cells were alive and capable of survival, minute differences in the way the tumours were treated had effects on their virulence. Therefore it should be added that on some occasions 2 tumours were disaggregated on the same day with the same reagents and inoculated into different batches of mice. It was found that some of these pairs of tumours gave contrasting colonization results. This is evidence that the differences in tumour behaviour are not due to batch variations in the mode of cell disaggregation, and is the only control available for such experiments, in which there is no standard tumour inoculum.

Two important questions to which the answers are not yet available are, first, whether mammary-tumour cells form deposits in the lung in preference to other organs because they have some special predilection for this organ, or because this is the first capillary bed in which they are arrested, and secondly, whether, in the LCP group, the inoculated tumour cells are dead at the time of necropsy or still alive but dormant. Experiments are in progress on these topics. It is therefore premature to comment on whether the findings with cells from primary tumours in our model confirm or differ from those with cultured and cloned cells inoculated i.v. in which organ specificity of metastasis was reported (Brunson *et al.*, 1978). It is also not possible at present to say whether latent cells capable of producing further colonies are present in the organs of animals found to be LCP at 3 months.

Many investigators have used i.v. inoculation of tumour-cell suspensions as a model of metastasis, and much useful information has been obtained with this method (*e.g.*, Zeidman, 1961; Hagmar & Norby, 1973; Van den Brenk *et al.*, 1973; Weiss *et al.*, 1974. See also reviews by Fisher & Fisher, 1967; Fidler, 1976, 1978). In such previous studies the tumour cells were obtained from serially propagated tumour-cell lines, or from repeatedly transplanted tumours, for two main reasons: ease of ensuring a ready supply of tumour cells, and the belief that this would provide a standard tumour inoculum and allow comparison of results from different experiments. However, it is important to bear in mind that such cells may be modified by long exposure to abnormal selection pressures exerted by growth in unusual sites and conditions, and those from a given passage or culture generation may not be really comparable with those from other generations. Kripke *et al.* (1978) reported an alternative approach designed to circumvent some of these difficulties. They used cloned cell lines obtained from a U.V.-induced sarcoma of recent origin (Tumour 2237) which were inoculated i.v. Their work indicates that cells within this tumour differed in metastatic potential though no comparison was made between different primary tumours.

Our present work augments these previous studies in providing a new method for comparing and analysing behavioural properties of *primary* tumours while retaining the principal advantages of i.v. inoculation, that the time and circumstances of tumour dissemination can be defined by the investigator. The observation that there are two main categories of mammary tumours differing in their colonization potential presents an opportunity for studying factors affecting tumour dissemination, and the consistency of results in 80% of the groups of mice studied makes the model reliable for further work on this topic. Although the colonization potential of a given tumour is not known in advance, study of the variables affecting colonization potential is made feasible by removal of aliquots of the disaggregated cells before i.v. inoculation. Various properties of the disaggregated tumour cells can thus be examined or experimentally manipulated *in vitro* and subsequently correlated with the colonization behaviour of portions of the same sample *in vivo*.

The two approaches, using primary and transplantable tumours, are probably best suited to studying different questions: the former to comparing features of cells from tumours of similar origin but dissimilar colonization potential, and the latter to studying the effects of given variables on tumour cells of known colonization potential. It is possible that correlation of results obtained with each of these systems will yield more insights on the process of dissemination and colonization than each on its own.

We wish to thank Dr D. Connell and Professor A. J. S. Davies of the Institute of Cancer Research for invaluable help and discussions, and Professor Lajtha of the Paterson Laboratories, Christie Hospital, Manchester, for advice and encouragement.

We also wish to thank Mrs I. Fisher for her excellent work and patience during preparation of the manuscript, as well as Mrs M. Naylor and Mr W. Hinkes for skilled assistance with the illustrations.

The work was financed by grants from the Cancer Research Campaign and the Royal Society, whose help is gratefully acknowledged.

### REFERENCES

BODMER, W., TRIPP, M. & BODMER, J. (1967) Application of a fluorochromatic cytotoxicity assay to human leukocyte typing. *Histocompatibility Testing*, **2**, 341.

BRUNSON, K. E., BEATTIE, G. & NICOLSON, G. L. (1978). Selection and altered properties of brain-colonizing metastatic melanoma. *Nature*, **272**, 543.

DUNN, T. B. (1958) Morphology of mammary tumours in mice. In *Physiopathology of Cancer*. Ed. F. Homburger. London: Cassell & Co. Ltd. p. 38.

FIDLER, I. J. (1976) Patterns of tumour cell arrest and development. In *Fundamental Aspects of Metastasis*. Ed. L. Weiss. Amsterdam: North Holland. p. 275.

FIDLER, I. J. (1978) Tumour heterogeneity and the biology of cancer invasion and metastasis. *Cancer Res.*, **38**, 2651.

FISHER, B. & FISHER, R. (1967) Metastases of cancer cells. In *Methods in Cancer Research*, **1**, 243.

HAGMAR, B. & NORBY, K. (1973) Influences of cultivation, trypsinization and aggregation on the transplantability of melanoma B16 cells. *Int. J. Cancer*, **11**, 663.

KRIPKE, M. L., GRUYS, E. & FIDLER, I. J. (1978) Metastatic heterogeneity of cells from an ultra-violet light-induced murine fibrosarcoma of recent origin. *Cancer Res.*, **38**, 2962.

VAN DEN BRENK, H. A. S., SHARPINGTON, C. & ORTON, C. (1973) Macrocolony assays in the rat of allogeneic Y-P388 and W-256 tumour cells injected intravenously: Dependence of colony forming efficiency on age of host and immunity. *Br. J. Cancer*, **27**, 134.

WEISS, L., GLAVES, D. & WAITE, D. A. (1974) The influence of host immunity on the arrest of circulating cancer cells and its modification by neuraminidase. *Int. J. Cancer*, **13**, 850.

ZEIDMAN, I. (1961) The fate of circulating tumour cells. I. Passage of cells through capillaries. *Cancer Res.*. **21**, 38.